# Exploring the Secrets of Long Noncoding RNAs

**DOI:** 10.3390/ijms16035467

**Published:** 2015-03-10

**Authors:** Mingyang Quan, Jinhui Chen, Deqiang Zhang

**Affiliations:** 1 National Engineering Laboratory for Tree Breeding, College of Biological Sciences and Technology, Beijing Forestry University, Beijing 100083, China; E-Mails: MingyangQuan@bjfu.edu.cn (M.Q.); jinhuichen@bjfu.edu.cn (J.C.); 2 Key Laboratory of Genetics and Breeding in Forest Trees and Ornamental Plants, Ministry of Education, College of Biological Sciences and Technology, Beijing Forestry University, Beijing 100083, China

**Keywords:** lncRNAs, functional genomics, transcriptional regulation, post-transcriptional regulation, epigenetics, lncRNAs in plants

## Abstract

High-throughput sequencing has revealed that the majority of RNAs have no capacity to encode protein. Among these non-coding transcripts, recent work has focused on the roles of long noncoding RNAs (lncRNAs) of >200 nucleotides. Although many of their attributes, such as patterns of expression, remain largely unknown, lncRNAs have key functions in transcriptional, post-transcriptional, and epigenetic gene regulation; Also, new work indicates their functions in scaffolding ribonuclear protein complexes. In plants, genome-wide identification of lncRNAs has been conducted in several species, including *Zea mays*, and recent research showed that lncRNAs regulate flowering time in the photoperiod pathway, and function in nodulation. In this review, we discuss the basic mechanisms by which lncRNAs regulate key cellular processes, using the large body of knowledge on animal and yeast lncRNAs to illustrate the significance of emerging work on lncRNAs in plants.

## 1. Introduction to LncRNAs

Eukaryotic genomes produce transcripts in a wide range of sizes, from long protein-coding mRNAs to short noncoding transcripts; In humans, about 2% of transcripts have the capacity to encode protein, with the remainder considered to be noncoding RNAs (ncRNAs) [1]. The ncRNAs include housekeeping RNAs such as ribosomal, transfer, small nuclear, and nucleolar RNAs, and regulatory ncRNAs. The short regulatory ncRNAs include microRNAs (miRNAs), small interfering RNAs (siRNAs), and Piwi-associated RNAs [2]. Although most work on regulatory ncRNAs has focused on short ncRNAs, recent work has revealed the importance of long ncRNAs (lncRNAs), defined as ncRNAs of more than 200 nt in length. Although only a few lncRNAs have been characterized to date, these studies revealed that ncRNAs can regulate gene expression at the transcriptional, post-transcriptional, and epigenetic levels [3].

Research in animal systems showed that lncRNAs participate in many significant biological processes, such as X chromosome inactivation and genomic imprinting [1]. In addition, research on lncRNAs has applications in the treatment of disease, such as cancer and Alzheimer’s disease [3]. In contrast to work in animals, research on plants lncRNAs has lagged. However, the limited work has revealed that lncRNAs also have significant roles in plants. In this review, we describe the basic modes of action, regulatory mechanisms, and functions of lncRNAs in animals and use these descriptions to illuminate and offer new perspectives on the potential roles and key functions of lncRNAs in plants.

## 2. Characteristics of LncRNAs

**Figure 1:**
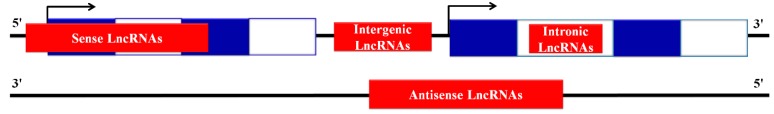
Four classes of lncRNAs. Blue indicates exons and white indicates introns; Black lines represent the coding and non-coding DNA strands. Red indicates lncRNAs, and arrows indicate the direction of transcription. Sense lncRNAs overlap with coding genes on the same strand. Antisense lncRNAs overlap with protein-coding genes on the opposite strand. Intronic lncRNAs occur completely within an intron. Intergenic lncRNAs occur between two genes.

LncRNAs can be divided into four rough categories according to their relationship to nearby protein-coding genes (Figure 1): Sense lncRNAs overlap with one or more exons of a transcript on the same strand, antisense lncRNAs overlap with one or more exons of a transcript on the opposite strand, intronic lncRNAs derive from an intron within another transcript, and intergenic lncRNAs occur in the interval between two genes on the same strand [1,4]. Both lncRNAs and short ncRNAs generally transcribe away from the 5' or 3' ends of genes, but most lncRNA transcripts originate near the promoters and the first exons or introns of genes [5]. Also, among mouse transcripts assigned as noncoding RNAs in the FANTOM2 set, which the transcripts have no complete ORFs or the longest ORFs are less than 100AA (amino acid), few (less than 2%) have open reading frames (ORFs) [6].

In addition to their length, the known lncRNAs have key differences from small ncRNAs, and some intriguing similarities to mRNAs. The small ncRNAs regulate gene expression by sequence-specific binding, but lncRNAs regulate gene expression through diverse mechanisms that remain unclear [7,8,9]. Most known lncRNAs are transcribed by RNA Polymerase II (RNAP II), similar to mRNAs. Some lncRNAs show additional similarities to mRNA, such as a 5' cap, 3' polyadenylated tail, and spicing, even though they have no potential to encode protein [10].

The expression of many ncRNAs varies specifically in different tissues [6,11] and both lncRNAs and mRNAs show varied cellular expression patterns and subcellular localizations. For example, the lncRNA Evf2 shows specific expression in the developing mouse brain [12]. Mercer *et al.* [12] analyzed over 800 lncRNAs from the Allen Mouse Brain Atlas, and found that lncRNAs occur in the nucleus or cell body, or are more concentrated in adult cerebellar Purkinje cells. Some lncRNAs show unique localization patterns, in novel subcellular compartments. For example, the Gomafu lncRNA occurs exclusively in nuclear speckles and Gomafu does not co-localize with any known nuclear compartment marker [13]. Also, many lncRNAs show specific expression at a particular developmental stage [14]; The majority of lncRNAs in mouse are specially expressed during embryonic stem cell differentiation and have a precise subcellular localization [12,15]. The lncRNA repertoire also seems to vary during evolution: Khaitovich *et al.* [16] found that, contrary to their expectation, the expression and divergence patterns of intergenic noncoding RNAs differed in three tissues from humans and chimpanzees.

Some small ncRNAs, like miRNAs, show high sequence conservation within the same family and regulate gene expression through sequence-specific binding to their targets. In contrast to the high conservation observed for small ncRNAs and many protein-encoding genes, lncRNA sequences tend to show weak sequence conservation. This can be explained by high rates of primary evolution, as lncRNAs may be frequent targets of positive selection, possibly reflecting the diversity and importance of lncRNA functions [17,18]. Additionally, compared to protein-coding genes, which have strict functional constraints and require a continuous ORF, lncRNAs can have weakly-conserved stretches of sequence that maintain functional domains and structures. For example, Xist, the lncRNA related to X-chromosome silencing, exhibits high conservation over short sections of its length [18]. Generally, the high conservation of promoter sequences [19] in lncRNAs and the low conservation of transcript sequences strongly indicate that lncRNAs have essential functions [20].

## 3. Mechanisms of Gene Regulation by LncRNAs

The widespread transcription of lncRNAs indicates that lncRNAs have diverse roles. Even though many of the mechanisms of lncRNA function remain to be discovered, lncRNAs are known to regulate gene expression in *cis* or in *trans* and function *via* transcriptional, post-transcriptional and epigenetic mechanisms, as described below.

### 3.1. Transcriptional Regulation

Different lncRNAs regulate transcription by diverse mechanisms, including affecting the transcription of adjacent genes in *cis*, interacting with RNAP II, cooperating with proteins, and acting as co-activators (Table 1).

**Table 1:** Examples of lncRNAs acting at the transcriptional level.

LncRNA	Target	Mechanism	Regulatory Effect	References
SRG1	*SER3*	The transcription of SRG1 suppresses the transcription of *SER3*.	NR	[21]
Upstream of *DHFR* ^a^	*DHFR*	LncRNA forms a triple helix in the promoter.	NR	[22]
B2	SINEs	Interacts with RNAPII to affect transcription during heat shock in mice.	NR	[23]
Alu	SINEs	Interacts with RNAPII to affect transcription during heat shock in human.	NR	[24]
Upstream of *CCND1* ^a^	*CCND1*	Induced by DNA damage signals, lncRNA binds with specific proteins to regulate target gene expression.	NR	[25]
HSR1	*HSPs*	HSR1 oligomerizes HSF1 to induce the expression of *HSPs* in response to heat shock.	PR	[26]
THRIL	*TNFα*	THRIL binds hnRNP-L to regulate the expression of *TNFα*.	PR	[27]
NRON	*NFAT*	LncRNA changes protein localization.	NR	[28]
ANRIL	*INK4B-ARF-INK4A* locus	ANRIL binds to the *INK4B* transcripts as a scaffold to recruit polycomb repressor to the *INK4B-ARF-INK4A* locus.	NR	[29]
Evf2	*Dlx2*	LncRNA acts as co-activator.	PR	[30,31,32]
LincRNA-p21	*P21*	LncRNA interacts with hnRNP-K as a coactivator to induce *p21* transcription.	PR	[33]

^a^ LncRNA is transcribed from the region upstream of the gene; NR indicates that the lncRNA negatively regulates the expression of the target gene, and PR indicates positive regulation of the target gene.

**Figure 2:**
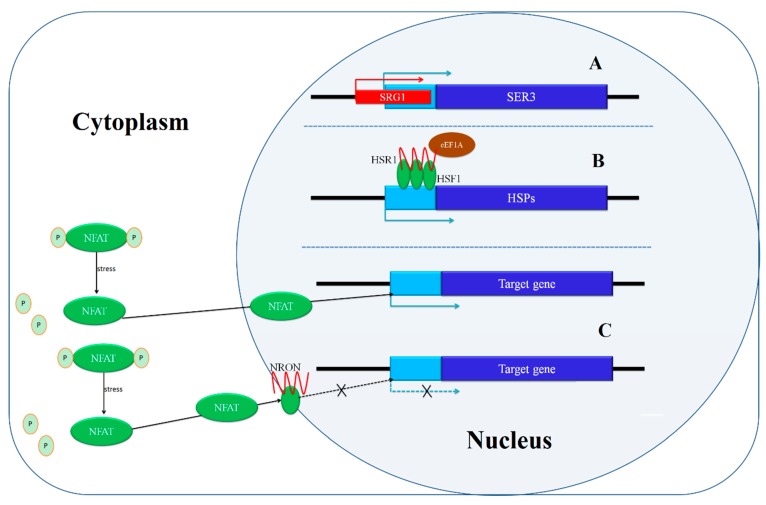
Transcriptional regulation of lncRNAs. Red indicates lncRNAs, blue indicates protein-coding genes, pale blue indicates promoter areas, and green indicates proteins. (**A**) The transcription of SRG1 interferes with the expression of *SER3*; (**B**) The lncRNA HSR1 promotes the trimerization of HSF1 after heat shock. The HSF1 trimers bind to the HSR1 promoter and up-regulate HSR1 expression. The complex of HSF1 trimers, HSR1, and translation elongation factor eEF1A promotes the expression of HSPs; (**C**) NFAT is highly phosphorylated in the cytoplasm. Outside stimuli induce dephosphorylation of NFAT, which then moves into the nucleus to activate gene expression. NRON forms a complex with other proteins to prevent NFAT from moving into the nucleus, thus preventing the activation of the target gene.

LncRNAs transcribed from the promoter region have a strong influence on the transcription of neighboring protein-coding genes and the mechanisms by which lncRNAs interfere with transcription depend on their location relative to the adjacent genes. For example, *Saccharomyces cerevisiae* *SER3* participates in the biosynthesis of serine; the transcript of lncRNA *SRG1* (SER3 regulatory gene 1) lies upstream of *SER3*, and the 3' end of SRG1 overlaps with the promoter of *SER3*. SRG1 regulates *SER3* in *cis* (Figure 2A); When SRG1 is expressed, the elongation of SRG1 suppresses the initiation of *SER3* transcription [21].

Some lncRNAs regulate gene expression by forming a triple helix in the promoter of the target gene. The *DHFR* (human dihydrofolate reductase) gene contains two promoters. The minor promoter upstream of *DHFR* encodes an lncRNA that forms an RNA-DNA triple helix with the sequences of the major promoter and interacts directly with TFIIB (transcription factor IIB), resulting in the dissociation of the pre-initiation complex in the major promoter and repression of *DHFR* [22]. Thus, interfering with transcription of the adjacent genes is one of the modes of actions of sense and intergenic lncRNAs.

LncRNAs can also interact with RNAP II to affect transcription. Short interspersed repeat elements (SINEs) produce Alu and B2 lncRNAs in human and mouse, respectively. When induced by heat shock, these lncRNAs bind to RNAP II to prevent the formation of active pre-initiation complexes and thus restrict the transcription of target genes [23,24]. Alu and B2 have a modular domain that inhibits the interaction of RNAP II and the target genes [34]. However, human scALU and mouse B1 lncRNAs lack the modular domain, which has a high affinity for RNAP II; these lncRNAs thus decrease the stability of complexes with the transcription factor TFIIF, and allow transcription to proceed. A chimeric B1 lncRNA containing the modular domain can form a complex with RNAP II to suppress the expression of target genes by interaction with TFIIF [35]. Thus, these two classes of lncRNAs block transcription in *trans* and the modular domain plays a key role in the stability of the complex of lncRNAs and RNAP II.

LncRNAs can also indirectly affect transcription in *cis* by binding specific proteins and either activating accessory transcription factors or promoting oligomerization of an activator protein. In human cell lines, DNA damage signals induce a class of lncRNA transcripts located in the 5' of the cyclin D1 (*CCND1*) promoter. These lncRNAs cooperate with the RNA-binding protein TLS (Translocated in Liposarcoma) in the promoter region of *CCND1*. TLS binds CBP (cAMP response element binding protein (CREB)) and p300 and is activated *via* an allosteric effect. Activated TLS interacts with histone acetyltransferases to inhibit the histone acetyltransferase activity of p300 and CBP, thus suppressing the expression of the target gene [25]. Also, the HSR1 (heat shock RNA 1) lncRNA can promote the trimerization of HSF1 (heat shock transcription factor 1) proteins after heat shock [26]. The HSF1 trimers bind to the *HSR1* promoter and up-regulate *HSR1* expression. The complex of HSF1 trimers, *HSR1*, and translation elongation factor eEF1A induces the expression of HSPs (heat shock proteins) to initiate the cell protection response [26] (Figure 2B). LncRNA THRIL (TNFα and hnRNPL related immunoregulatory lncRNA) forms a complex with hnRNP-L, a component of hnRNP (heterogenous nuclear ribonucleoprotein). The THRIL-hnRNPL complex regulates the expression of TNFα by binding to its promoter. THRIL is essential for complex formation with hnRNP-L and the expression of TNFα, indicating the vital roles in the immune response [27]. The expression of these lncRNAs responds to environmental conditions and these lncRNAs act as intermediaries among transcription factors and related proteins. Thus, lncRNAs can positively affect their target genes as signaling molecules and essential elements in response to outside stimuli.

LncRNAs can also affect protein localization to regulate gene expression. For example, the NRON(noncoding repressor of nuclear factor of activated T cells (*NFAT*)) lncRNA regulates intracellular trafficking. Recent research showed that in unstressed conditions, NFAT is highly phosphorylated and localizes to the cytoplasm. Outside stimuli induce an increase in cytoplasmic calcium, causing a calmodulin-dependent phosphatase to dephosphorylate NFAT, which then moves into the nucleus to activate gene expression. NRON forms a complex with three kinds of NFAT kinases and an IQ (the first two amino acids of the motif are isoleucine and glutamine) motif-containing GTPase-activating protein to prevent NFAT from moving into the nucleus, thus preventing the activation of NFAT target genes [28] (Figure 2C). In addition, a number of lncRNAs can recognize signals, such as DNA damage, and bind to a target locus, where they function as a scaffold to assemble effector molecules. ANRIL is induced by DNA damage signal and binds to the *INK4B* transcript as a scaffold. ANRIL binds in INK4B-ARF-INK4A locus, which can be recognized as signal molecule, and recruits the polycomb repressor complex; thus, the transcription of the genes at this locus is repressed [29]. According to the mechanism, recognition of the target sites requires high conservation over short sections of the lncRNAs or the particular secondary structures of lncRNAs.

LncRNAs can also act as co-activators to control transcription. The vertebrate *Dlx* homeodomain genes affect neuronal development and migration. Two ultra-conserved intergenic enhancers are located between *Dlx5* and *Dlx6*, and the Evf2 lncRNA transcribes from one of these enhancers. *Dlx2* can be activated *via* forming a stable complex with *Evf2*; this complex recruits the transcription factor MeCP2 (methyl CpG binding protein 2) to the other enhancer [30], increasing the transcription of *Dlx5/6*. These events regulate the development of γ-aminobutyric acid neurons and the brain excitatory neural network [31,32]. *p53* acts as an important tumor suppressor gene in the response to DNA damage. LincRNA-p21, one of the lncRNAs activated by p53, plays a role in the p53-regulated apoptosis pathway [36]. Dimitrova *et al.* found that lincRNA-p21 activates the expression of the adjacent gene *p21.* They found that lincRNA-p21 interacts with hnRNP-K as a co-activator to induce the *p21* transcription in *cis*, thus promoting the expression of polycomb target genes. The expression of *p21* decreased after loss of lincRNA-p21; Thus, although hnRNP-K was present, the expression of the polycomb target genes was down-regulated and the chromatin state of the genes was altered [33]. This research suggests that lincRNA-p21 influences global gene expression as a co-activator of *p21* expression.

Thus, lncRNAs can affect gene expression by binding specific regions in the target genes and cooperating with proteins or transcriptional elements to regulate transcription. The diverse function of lncRNAs depends on not only their specific structures and sequences, but also on their binding to transcriptional elements.

### 3.2. Post-Transcriptional Regulation

LncRNAs also function at the post-transcriptional level, acting by diverse mechanisms and likely functioning *via* mediators, such as miRNAs, including functioning as the precursors of small RNAs, and acting with miRNAs to regulate mRNA turnover. In addition, lncRNAs can also affect translation by influencing the alternative splicing of pre-mRNAs and can regulate mRNA stability in *trans*, which requires the lncRNA and the target gene to have complementary sequences (Table 2).

**Table 2:** Examples of lncRNAs acting at the post-transcriptional level.

Biological Process	LncRNA	Target	Mechanism	References
Splicing	MALAT1	SR proteins	The lncRNA affects pre-mRNA splicing to produce a variety of proteins.	[37,38]
miRNA regulation of translation	Linc-MD1	*MEF2C* and *MAMAL1*	LncRNA interacts with miRNA-133 and miRNA-135 to regulate their activity and control the translation of *MEF2C* and *MAMAL1*.	[39,40]
	Uc.283+A	pri-miR-195	Uc.283+A prevents pri-miR-195 from processing into mature miRNA.	[41]
mRNA turnover	BACE1-AS	*BACE1* mRNA	LncRNA increases the stability of the *BACE1* mRNA by perfect base pairing with *BACE1* mRNA.	[42]
	1/2sbsRNA	mRNA contains Staufen-1 binding sites and *Alu* sequence	Imperfect base pairing of lncRNA and target mRNA leads to the decay of target mRNA.	[43,44]

LncRNAs occur in the cytoplasm and nucleus [45]. Nuclear lncRNAs can affect mRNA capping, polyadenylation and pre-mRNA splicing. In eukaryotes, the production of diverse proteins depends on the variety of pre-mRNA splicing, and lncRNAs can affect splicing, including alternative splicing (AS) [46,47,48]. For example, the lncRNA MALAT1 (metastasis-associated long adenocarcinoma transcript 1), located in nuclear speckles, interacts with and changes the distribution of splicing factors, such as SR (serine/arginine-rich) proteins. Depletion of MALAT1 affects the abundance, location, and activity of SR proteins and changes the AS of a series of pre-mRNAs [37]. In *Arabidopsis*, NSRs (nuclear speckle RNA-binding proteins) act as AS regulators to interact with their alternatively spliced mRNA targets and ASCO-lncRNAs (AS-competitor lncRNAs). The expression of lncRNAs alters the splicing pattern of mRNAs regulated by NSRs and competes with AS targets to bind with NSRs. Several biological processes, such as the formation of lateral roots in plants, involve the regulation of gene expression by lncRNAs, which hijack regulators of spicing machinery [38]. Thus, lncRNAs regulate crucial pathways in plants and animals by various splicing-related mechanisms.

Some lncRNA sequences have been annotated as precursors of small RNAs. A series of small RNAs have been mapped to some of the regions along lncRNA transcripts and Dicer or Drosha enzymes can produce small ncRNAs, such as miRNAs and piRNAs, from lncRNAs [49]. Also, antisense lncRNAs transcribed from pseudogenic and natural antisense transcripts can bind a specific mRNA to form duplexes, which are then degraded into endogenous siRNAs that have the potential to regulate the specific mRNA [50,51].

Also, lncRNAs can interact with miRNAs to regulate miRNA activity and thus control the translation of target mRNAs. Linc-MD1 (long intergenic noncoding RNA-muscle differentiation 1), a muscle-specific lncRNA, binds to miRNA-133 and miRNA-135 to regulate muscle differentiation. In the cytoplasm, Linc-MD1 acts as an endogenous competitor RNA to prevent these miRNAs from contacting their target mRNAs, acting as a molecular sponge to “soak up” miRNA-133 and miRNA-135 and thus promote the expression of genes that encode two transcription factors related to muscle differentiation. Depletion and overexpression of Linc-MD1 retard and accelerate muscle differentiation, respectively [39]. As the Linc-MD1 primary transcript contains a pri-miRNA-133b sequence, it also may function as a miRNA precursor. The balance between miRNA-133 biogenesis and Linc-MD1 is controlled by the myogenesis-related RNA-binding protein HuR (human antigen R) [40]. The myoblasts of patients with Duchene Muscular Dystrophy, a severe myopathy, have reduced levels of Linc-MD1 [39]. In addition, lncRNA Uc.283+A has an ultra-conserved transcript, which is complementary with the lower stem region of pri-miR-195. Regulation of the complementarity between Uc.283+A and pri-miR-195 prevents pri-miR-195 processing into mature RNA [41]. Notably, the ultra-conserved regions of lncRNAs have vital roles to their regulatory effects. Thus, some classes of lncRNAs are processed into small RNAs and interact with small RNAs to affect gene expression. The complex network of ncRNAs and mRNAs represents a new layer of gene regulation in animals and plants.

**Figure 3:**
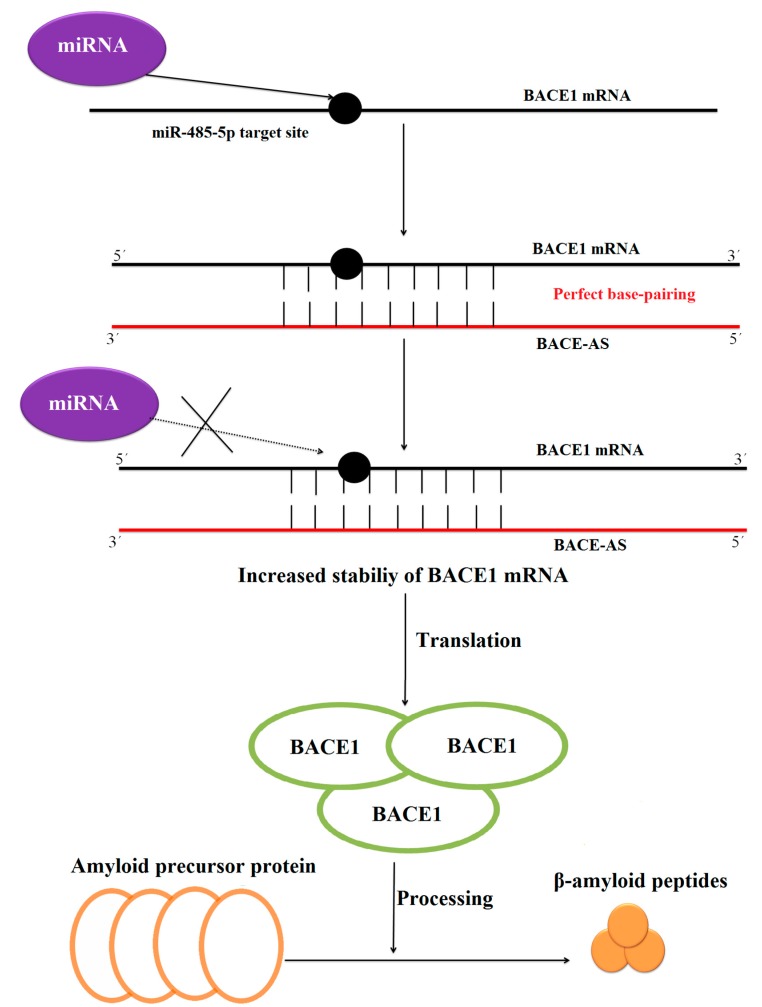
The model of BACE1-AS lncRNAs. Black indicates BACE1 mRNA, red indicates BACE1-AS lncRNA, purple indicates miRNA, and orange and green circles indicate proteins. The BACE1-AS lncRNA can form perfect base pairs with the BACE1 mRNA for about 100 nucleotides, which includes the target site for miR-485-5p. The increased stability of BACE1 mRNA leads to increased abundance of BACE1 protein. BACE1 proteins proteolytically process the amyloid precursor protein into toxic substances such as β-amyloid peptides associated with Alzheimer’s disease.

LncRNAs can act directly on their target mRNAs to positively or negatively affect translation. LncRNAs can affect mRNA stability, as base pairing of lncRNAs and mRNAs can protect mRNA from degradation or accelerate its turnover [52]. For example, the enzyme BACE1 (β-secretase-1) affects Alzheimer’s disease by proteolytically processing the amyloid precursor protein into poisonous substances such as β-amyloid peptides [42,53] (Figure 3). The BACE1-AS (*BACE1*-antisense transcript) lncRNA plays a vital role in the stability of *BACE1* mRNA [42], and the increased stability of *BACE1* mRNA results in increased BACE1. BACE1-AS overlaps with the *BACE1* mRNA by 100 nucleotides, where the mRNA and lncRNA can form perfect base pairs; this overlap also includes a target site for miR-485-5p. BACE-AS and miR-485-5p compete to bind the *BACE1* mRNA. MiR-485-5p decreases *BACE1* mRNA stability, and BACE1-AS increases *BACE1* mRNA stability; the perfect base pairing with the lncRNA protects the *BACE1* mRNA from degradation by the miRNA [54]. The lncRNAs BC200 (brain cytoplasmic RNA 1) and BACE1-AS are associated with Alzheimer’s disease [42,55]; Thus, regulation by these lncRNAs may play a key role in human disease.

Many routes lead to mRNA decay, including NMD (nonsense-mediated mRNA decay). The RNA binding protein Staufen1 binds to the NMD factor Upf1 (up-frameshift protein 1) and brings Upf1 to the 3'UTR of the mRNA, causing mRNA decay [43]. Imperfect base pairing of the lncRNA 1/2sbsRNA (half Staufen1 binding site RNA) to the 3'UTR of mRNA from *Alu* element forms binding sites for Staufen1. This process promotes the combination of Staufen1 and the *Alu* mRNA, and thus stimulates mRNA decay [44]. The examples mentioned above demonstrate the flexibility of lncRNAs in regulating post-transcriptional events.

### 3.3. LncRNAs and Epigenetic Regulation

Epigenetic regulation plays a vital, emerging role in gene regulation and involves DNA methylation, histone modification, X-chromosome inactivation, imprinting, chromosome dosage compensation, and other aspects. Here, we introduce some of these to illustrate the regulatory roles of lncRNAs in epigenetic mechanisms (Table 3).

**Table 3:** Examples of lncRNAs involved in epigenetic regulation.

Biological Process	LncRNA	Comments	References
DNA methylation	Khps1a	Methylation levels decrease when Khps1a binds, thus increasing the expression of related genes.	[56]
	ecCEBP	Binds DNMT1 to control the methylation level of target genes.	[57]
X-chromosome inactivation	Xist	Spreads to the Xi to maintain XCI.	[58]
	Tsix	Antagonizes Xist to participate in XCI by competing for PRC2 with RepA.	[58]
	Xite	Acts as an enhancer of Tsix to affect XCI.	[59]
	RepA	Recruits PRC2 to activate the transcription of Xist.	[60]
	Jpx	Functions as a positive regulator to activate Xist.	[61]
	XACT	A human-specific lncRNA that coats the active X chromosome.	[62]
Genome imprinting	H19	Expresses the maternal allele and regulates the expression of *Igf2r* by affecting the methylation status of the ICR.	[63,64,65]
	Kcnq1ot1	Kcnq1ot1 expression depends on the methylation status in ICR, and it expresses paternally.	[66,67]
	Air	Expressed due to the lack of methylation of ICR in the *Igf2r* domain on the paternal allele.	[68]

#### 3.3.1. LncRNAs Involved in DNA Methylation

During development, the establishment and maintenance of DNA methylation lead to the modification of gene expression. DNA methylation typically occurs in CpG islands in the promoter region of a gene. The CpG island in the tissue-dependent differentially methylated region of sphingosine kinase1 (*Sphk1*) can produce several lncRNAs, including the 1290 nt Khps1a (*Rattus norvegicus Sphk1a* antisense transcript). Binding of Khps1a to three CC(A/T)GG sites reduces the methylation of the CpG island and increases the expression of the oncogene *Sphk1* [56]. Recent work showed that a series of lncRNAs depend on their association with DNA methytransferase 1 (DNMT1) to regulate DNA methylation. The ecCEBP (extra-coding RNA) lncRNA arises from the *CEBPA* gene as a non-polyadenylated, sense transcript that initiates about 2 kb from the *CEBPA* transcription start site and extends 3 kb along the *CEBP* mRNA. The ecCEBP lncRNA binds with DNMT1 and inhibits methylation of the *CEBPA* locus [57]. This intriguing model involves a mechanism by which a non-polyadenylated transcript enhances function at distal regulatory elements to regulate the DNA methylation levels in enhancer sites. Other methyltransferases may also interact with lncRNAs to regulate DNA methylation [69].

#### 3.3.2. X-Chromosome Inactivation

X chromosome inactivation (XCI) and genomic imprinting in mammalian cells balance the gene expression level of the X chromosome between males and females [1]. In XCI, dosage compensation inactivates one of the X chromosomes in females *via* the X inactivation center (Xic). The Xist lncRNA transcribes from the Xic and eventually covers the entire inactive X chromosome (Xi). Chromatin remodeling, histone modification, and DNA methylation then inactivate gene expression on Xi [70].

The Xic also produces other lncRNAs, including Tsix, RepA, Xite, and Jpx. Tsix is an antisense transcript of *Xist* [58]. The 1.6 kb RepA transcript is derived from the 5' end of *Xist* and may activate *Xist* expression. *Xist* expression is controlled by Tsix and RepA, which compete for recruiting the Polycomb Repressor Complex 2 (PRC2) [58]; They act antagonistically on *Xist*, with RepA acting positively to recruit PRC2 to the *Xist* promoter, and Tsix acting negatively [71] to preventing RepA-PRC2 from loading and thus hindering the initiation of XCI. RepA recruits PRC2 to the promoter of *Xist*, and Xist co-transcriptionally recruits PRC2 *via* RepA to form a complex [60]. Loss of Tsix and induction of Jpx induce the expression of *Xist*, which permits RepA-PRC2 loading onto chromatin. Jpx also functions as a positive regulator to activate *Xist* expression [61] and Xite, as an upstream enhancer, affects XCI by regulating Tsix [59].

**Figure 4:**
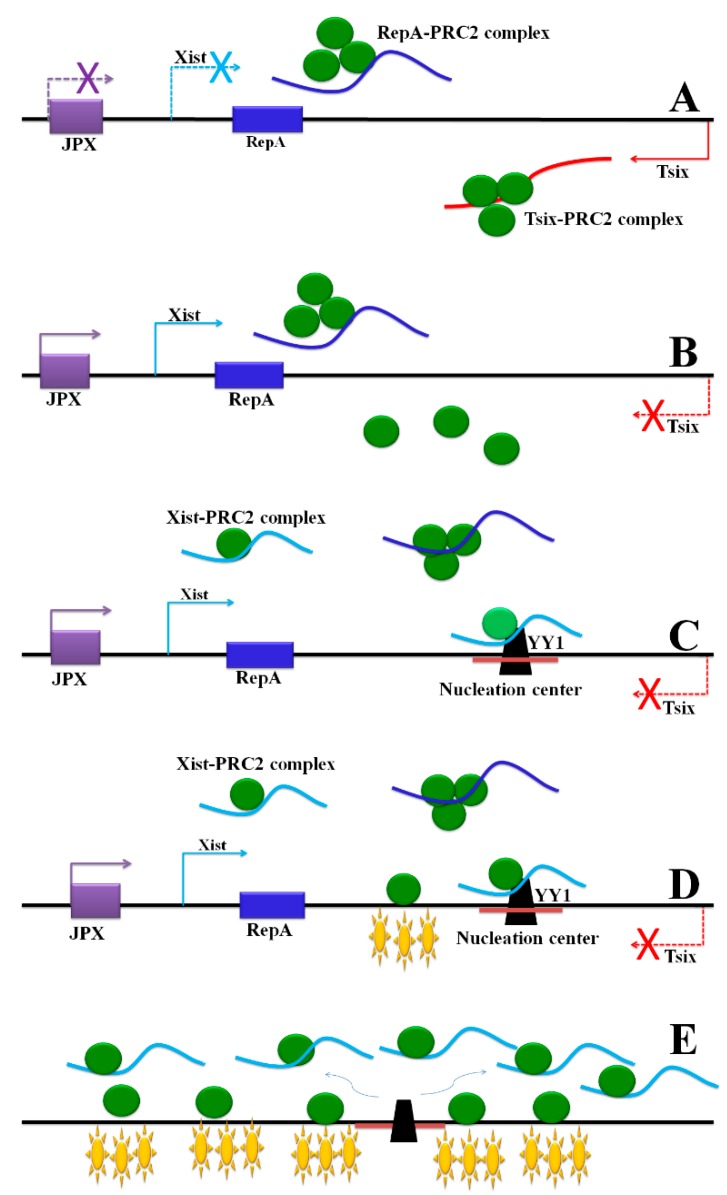
The steps in initiation of X chromosome inactivation. The arrows (red, blue and purple) represent the transcriptional direction of lncRNAs. The solid arrows (red, blue and purple) indicate lncRNAs are expressed in corresponding loci, and the dotted arrows (red, blue and purple) with forks indicate the lncRNAs stop to transcribe in these loci. (**A**) Both Tsix and RepA recruit PRC2 to form a complex; Tsix prevents the RepA-PRC2 complex from loading onto chromatin; (**B**) Induction of JPX and loss of Tsix enable Xist expression; (**C**) RepA recruits PRC2 to the promoter of Xist; Xist co-transcriptionally recruits PRC2 to form a complex. YY1 binds with the complex in the nucleation center; (**D**) PRC2 binds to the chromatin and generates H3K27me3 (purple asterisks); (**E**) The Xist-PRC2 complex spreads along the chromosome from the nucleation center. PRC2 distributed on the chromosome generates H3K27me3.

The YY1 transcription factor only binds to the Xi nucleation center, which bridges the Xist-PRC2 complex to bind to the Xi. The Xist-PRC2 complex co-transcriptionally binds to the YY1-based nucleation center in the first exon of *Xist*. Here, YY1 functions as a bridge that brings the Xist-PRC2 complex to the Xi [72], where PRC2 increases H3K27me3 [58]. The Xist-PRC2 complex spreads along the future Xi and methylation occurs over the entire chromosome [73] (Figure 4). A recent study illuminated the mechanism of Xist localization during the initial and maintenance phases of XCI. Xist is transferred to distal sites along the X chromosome that are spatially proximate to the Xist transcription locus, instead of to specific sequences. Thus, Xist spreads broadly across the X chromosome without focal binding sites [74].

Two more discoveries have enriched our understanding of XCI. The novel lncRNA XACT (X active coating transcript) is produced in human pluripotent stem cells. XACT can coat the active X chromosome in the absence of Xist expression; this is an additional mechanism acting specifically in humans [62]. In addition, Jarid2 functions as an important co-factor of PRC2 to mediate Xist-induced PRC2 targeting. Jarid2 is essential for recruiting PRC2 efficiently, but first recruits PRC2 independently and promotes the process of PRC2 targeting to the X chromosome during XCI [75].

#### 3.3.3. Genomic Imprinting

For an imprinted gene, the alleles from different parents express differently in the offspring. Some genes show transcriptional activation of the paternal allele, and silencing of the same allele from the maternal lineage. Others genes act oppositely, with the maternal allele active and the paternal allele silenced. Analogous to the Xic governing XCI, the ICR (imprinting control region) governs imprinting of the locus. The lncRNA H19 regulates the expression of several imprinted genes involved in growth control in mice. In the regulation of the imprinted gene *Igr2*, the *H19* and *Igf2* loci share the same enhancer, but *Igr2* expresses only from the paternal allele and H19 expresses only from the maternal allele. In the maternal allele, the ICR is unmethylated, which leads to the multifunctional transcription factor CTCF (CCCTC-binding factor) binding to the ICR, preventing the downstream enhancer from interacting with *Igf2* and allowing *H19* to access the enhancer. In the paternal allele, methylation of the ICR extends to the promoter of *H19*, which silences the expression of *H19* and prevents CTCF from binding to the ICR. Thus, the enhancer can activate expression of *Igf2r* from the paternal allele [63,64,65]. The key to this mechanism depends on the CTCF binding region and may function in the regulation of other imprinted genes.

In some cases, lncRNAs silence the imprinted gene in *cis*. LncRNAs overlap with the adjacent imprinted genes, and influence gene transcription by causing transcriptional interference with the promoter. For example, the Kcnq1ot1 lncRNA expresses in the imprinted Kcnq1 domain, from its promoter in the ICR. The expression of Kcnq1ot1 lncRNA or nearby imprinted genes depends on the methylation status of the ICR [66,67]. For the Igf2r domain, *Igf2r* and the locus producing the Air lncRNA transcribe in opposite directions. The ICR is located in the second intron of *Igf2r*, which contains the *Air* promoter; Thus the ICR and the *Air* promoter overlap slightly. ICR is unmethylated on the paternal allele, which leads to expression from the *Air* locus, partly by removing RNAP II from the promoter [65]. The *Air* transcript covers the *Igf2r* promoter, so expression of *Air* inhibits the expression of *Igf2r*. On the maternal allele, the methylated ICR stops the transcription of *Air* and allows the expression of *Igf2r* [68]. Accumulation of Air mediates the imprinting of the *Slc22a2* and *Slc22a3* loci, silencing the paternal allele in *cis* [67,68,76].

## 4. LncRNAs in Plants

Large scale, full-length sequencing of a cDNA library in mouse identified many lncRNAs [77]; This research set off a wave of studies in mammals and plants. Even though a number of plant lncRNAs have been identified and some mechanisms have been brought to light, many issues on plant lncRNAs remain to be addressed (Table 4). As with other phenomena, particularly epigenetics, emerging research in plants will doubtless eventually inform research in animal systems.

### 4.1. The LDMAR LncRNA Affects Male Fertility in Rice

The rice (*Oryza sativa*) LDMAR (long day specific male fertility associated RNA) lncRNA functions to regulate PSMS (photoperiod-sensitive male sterility) and provides an important tool for generating hybrid rice, as the *pms3* mutant lines are sterile under long-day conditions and fertile under short-day conditions. Under long-day conditions, normal pollen development requires the 1236-nucleotide LDMAR transcript [78]. Although the *LDMAR* mRNA has a small open reading frame, alteration of the ATG start codon did not affect LDMAR function, and expression of only the open reading frame also showed no effect on male sterility, indicating that LDMAR indeed acts as an lncRNA [78]. A natural, single-nucleotide mutation of LDMAR in *pms3* male-sterile varieties altered the secondary structure of LDMAR by perturbing a predicted stem-loop. The *pms3* line showed increased methylation in the promoter region of *LDMAR*, and this methylation likely occurs by RNA-mediated DNA methylation caused by the small interfering RNA Psi-LDMAR, produced from a sense transcript from the *LDMAR* region and targeting the *LDMAR* promoter [79]. The decrease in transcription of *LDMAR* resulting from increased methylation of the promoter may cause premature programmed cell death in developing anthers, and thus result in PSMS. However, Zhou *et al.* [80] identified a 21-nt small RNA, osa-smR5864w, which is altered by the same polymorphism in *pms3* and also affects male fertility. Osa-smR5864w derives from a 136-nt precursor, indicating that LDMAR may be processed to smaller forms that serve as siRNA precursors. Also, RNA-directed DNA methylation (RdDM) affects PSMS in rice. Therefore, the precise interactions between this lncRNA and siRNAs, the function of the other transcripts from this locus, and the mechanism by which LDMAR and siRNAs affect anther development, remain to be clarified.

### 4.2. LncRNAs Direct Protein Re-Localization in Symbiosis

Enod40, one of the first lncRNAs identified in plants, functions in regulation of symbiotic interactions between leguminous plants and soil bacteria [81,82]. Enod40 shows high sequence conservation in legumes and non-legume species, such as rice, [83], but lacks an open reading frame in non-legume species [84,85]. However, Enod40 RNA encodes two short peptides, of 12 and 24 amino acids in soybean and 13 and 27 amino acids in *Medicago truncatula* [84,85]. The peptides encoded by soybean Enod40 bind sucrose synthase, suggesting a function for Enod40 in regulation of sucrose utilization in nodules [84]. In *M. truncatula*, biological activity of Enod40 is related to the translation of the two peptides [85].

Several lines of evidence support the hypothesis that Enod40 function requires its RNA molecule, instead of the short peptides. First, Enod40 has a stable RNA secondary structure. In leguminous species, five conserved domains exist in the Enod40 transcripts; at least two of these domains exist in all Enod40 homologues and are absolutely conserved [83,86]. Also, the functional secondary structure of Enod40 is important. An altered Enod40 that lacks the RNA structural elements but retains the proper number of short peptides showed decreased function in the formation of nodules and the stimulation of cortical cell division in *M. truncatula* [85]. Moreover, one of the two short peptides is not conserved. Remarkably, the Enod40 RNA structural elements have remained more conserved than the encoded peptides [63]. Thus, the evidence supports the hypothesis that Enod40 acts as an ncRNA.

Enod40 can affect protein localization. MtRBP1 (*M. truncatula* small nodulin acidic RNA-binding protein 1) is an RNA-binding protein that is expressed constitutively. During nodulation in *M. truncatula*, Enod40 interacts directly with MtRBP1 and relocalizes MtRBP1 from nuclear speckles to cytoplasmic granules [87]. This re-localization of MtRBP1 only occurs in Enod40-expressing plants, and the same phenomenon occurs in the plants with impaired translation of Enod40 peptides [87]. The above observations suggest that the Enod40 RNA, rather than the peptides encoded by Enod40, is responsible for the re-localization of MtRBP1. This may also indicate that, as in animals, lncRNAs can re-localize proteins in plants.

**Table 4:** Examples of lncRNAs in plants.

LncRNA	Biological Function	Biological Processes Regulated by LncRNAs	References
LDMAR	Regulates photoperiod-sensitive male sterility.	Photoperiod-sensitive male sterility	[78,79,80]
Enod40	Encodes two short peptides and an ncRNA with functional secondary structure.	Sucrose utilization in nodules and nodulation in *Medicago truncatula*	[81,82,83,84,85]
IPS1	Competes with *PHO2* to interact with miR399, and acts as a miRNA target.	Phosphate balance	[88]
COLDAIR	Recruits PRC2 to silence *FLC* and is important to sustain the stability of *FLC* state and vernalization response.	Vernalization	[89,90]
COOLAIR	Functions with other vernalization-related elements in regulating *FLC* expression.	Vernalization	[91]
ASL	Regulated by AtRRP6L to maintain the level of H3K27me3.	Vernalization	[92]
HID1	Acts as a positive regulator in photomorphogenesis.	Photomorphogenesis	[93]

### 4.3. LncRNAs Function as miRNA Target Mimics

Work in *Arabidopsis thaliana* identified a mechanism of lncRNA function that resembles the miRNA sponges in animal systems [88], showing that some plant lncRNAs can interact with miRNAs as competitors and function as miRNA target mimics. Plants have a complicated mechanism that regulates phosphate uptake to maintain the balance of phosphate and meet the plant’s needs for growth and development. miRNAs have essential functions in the mechanisms regulating phosphate balance, and miR399 is highly expressed under phosphate starvation [94]. This miRNA targets *PHO2*, which encodes an E2 ubiquitin conjugase-related enzyme and is repressed by mRNA cleavage mediated by miR399. Low activity of PHO2 leads to increased expression of two root-specific phosphate transporter genes, leading to increased phosphate uptake [94]. Phosphate starvation also induces the lncRNA IPS1 (Induced by Phosphate Starvation 1), which has a 23-nt conserved domain in different plant species, such as *A.* *thaliana* and *M. truncatula*, and a 23-nt motif with partial complementarity to miR399, with a 3-nt central mismatch [88]. The mismatch of miR399 and IPS1 overlaps with the region where miR399-mediated cleavage occurs. Thus, the imperfect base pairing of miR399 and IPS1 prevents miR399-mediated cleavage of IPS1. IPS1 as a non-cleavable target mimic and competitor to *PHO2*, can weaken the miR399-mediated repression of *PHO2* [88]*.* This mechanism in which lncRNAs act as target mimics to compete with genes, a similar strategy to the miRNA sponge strategy, offers insights on the interactions of miRNAs and lncRNAs in plants. The target mimic mechanism, which has therapeutic applications in treatment of human disease, may also provide a useful tool in plants, as the use of lncRNAs as target mimics to inhibit miRNAs in regulating the expression of their target genes may have both research and agronomic applications.

### 4.4. Plant LncRNAs in the Regulation of Flowering

In *A. thaliana*, multiple pathways regulate expression of the floral inhibitor *FLC* (*FLOWERING LOCUS C*) to fine-tune flowering time [95]; For example, inhibition of *FLC* by vernalization promotes flowering [96]. Epigenetic regulation by histone modifications and a set of lncRNAs plays a crucial role in regulating the expression of *FLC*. Several modifications of histone 3 affect *FLC* expression, such as the methylation of H3K4 and H3K27. In *FLC* chromatin, H3K4 methylation leads to a permissive chromatin state, which is required for *FLC* expression. By contrast, H3K4 demethylation contributes to *FLC* repression [97]. H3K27me3 is also required for *FLC* silencing [89].

**Figure 5:**
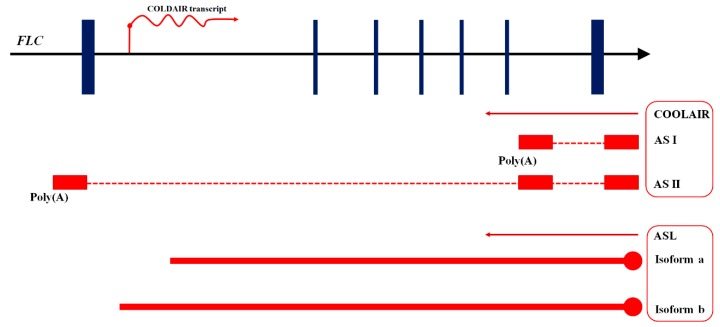
LncRNAs from *FLC*. The blue bars indicate the exons of *FLC* sense transcripts. The arrows represent the direction of transcription. The red arrows indicate the direction of transcription. The red circular endpoints correspond to the 5' cap of lncRNAs. COLDAIR is transcribed from the first intron of FLC in the sense direction relative to *FLC* mRNA. COOLAIR and ASL transcripts are transcribed in the antisense direction. AS I and AS II, two antisense transcripts of COOLAIR, are transcribed in the antisense direction relative to *FLC* mRNA. Red boxes correspond to AS I and II exons, and dotted red lines correspond to the spliced regions of AS I and II. AS I has a proximal polyA in the sixth intron of *FLC*, and AS II has a distal polyA that overlaps with the promoter region of *FLC*. ASL is an antisense lncRNA with two isoforms produced by alternative spicing. The two red bold lines indicate the two isoforms of ASL. They transcribe from the same promoter as COOLAIR and span the first intron of *FLC*.

COLDAIR and COOLAIR, two classes of lncRNAs transcribed from *FLC*, participate in epigenetic silencing of *FLC*. COOLAIR antisense lncRNAs, which transcribe from the 3' end of *FLC*, include two alternatively polyadenylated isoforms, AS I and AS II. AS I has a proximal poly(A) in the sixth intron of *FLC*, and AS II has a distal poly(A) in the promoter region of *FLC* (Figure 5). The two isoforms affect H3K4 demethylation to silence *FLC* [91,97,98]. They act at the transcriptional level, unlike other lncRNAs in plants. The autonomous flowering pathway modulates the transcription of *FLC* by altering COOLAIR splicing. The increased proximal poly(A) of COOLAIR leads to enhanced demethylation of H3K4 in the gene body and reduces transcription of *FLC*, generating a positive feedback mechanism that reinforces the proximal polyadenylation of COOLAIR [99,100]. The distal polyadenylation depends on the expression of *FLC* [99,101]. In addition, the R-loop in the promoter region of COOLAIR also inhibits the expression of COOLAIR in *Arabidopsis*. Differential stabilization of the R-loop depends on the binding of the homeodomain protein AtNAD, thus influencing the transcription of COOLAIR [102].

Vernalization induces COOLAIR expression and represses *FLC* expression. The expression of COOLAIR increases earlier than other vernalization markers, such as *VIN3* (*VERNALIZATON INSENSITIVE 3*) [91]. Insertional mutants in the promoter of *COOLAIR*, however, do not cause a reduction of *FLC* expression, as the promoter and the first exon of *FLC* are sufficient for the initiation of *FLC* repression [103]. This indicates that COOLAIR functions along with other vernalization-related elements to regulate the expression of *FLC*. Recent results show that COOLAIR is associated with genomic DNA in the *FLC* locus and plays a vital role in accelerating the transcriptional repression of *FLC* during cold exposure. COOLAIR regulates H3K36me3, which has important effects on chromatin state, but COOLAIR and H3K27me3 function independently, consistent with the antagonistic interaction between H3K27me3 and H3K36me3. Thus, these findings indicate that COOLAIR functions in coordinating switching of chromatin states [95]. Also, this illustrates that plant antisense lncRNAs can affect the transcription of genes by mechanisms similar to those of animal lncRNAs.

The COLDAIR intronic lncRNA has a 5' capped structure and is transcribed from the first intron of *FLC*, in the same direction as *FLC* (Figure 5). Vernalization transiently induces the expression of COLDAIR, but COLDAIR expression peaks later than COOLAIR expression. COLDAIR directly binds CLF (CURLY LEAF), one of the components of PRC2. COLDAIR recruits PRC2 to silence the *FLC* locus by increasing H3K27me3 levels [89]. When *COLDAIR* is suppressed, the H3K27me3 level decreases, the response to vernalization decreases, and *FLC* expression decreases when these plants return to warm temperatures. The repression of *FLC* cannot be maintained in mutants affecting the components of PRC2. The above observations indicate that COLDAIR has vital functions in the stability of the *FLC* silenced state and the vernalization response [89,90].

Work in *A. thaliana* identified a novel antisense transcript termed ASL (Antisense long), which is transcribed from the *FLC* locus in wild-type plants [92]. ASL has the same promoter as AS I and AS II, and the transcript of ASL overlaps with the 5' region of AS I and AS II; however, ASL differs from AS I and AS II. Alternative splicing produces two isoforms of ASL, 2236 and 2536 nt long (Figure 5). Moreover, ASL is capped, non-polyadenylated, and synthesized by RNAP II. Thus, the ASL antisense lncRNA differs from COOLAIR AS I and AS II, which are polyadenylated [92]. ASL may be responsible for maintenance of H3K27me3, as ASL spans the first intron of *FLC* and overlaps with COLDAIR, which functions in establishing and sustaining the levels of H3K27me3. RRP6 (Ribosomal RNA-processing Protein 6) is a subunit of the exosome, a nuclear complex with 3' to 5' exoribonuclease activity. The AtRRP6L (*A. thaliana* RRP6-Like) proteins, homologs of RRP6, are responsible for ASL synthesis, because the mutants of AtRRP6L proteins have little or no ASL transcript. Moreover, AtRRP6L1 physically interacts with ASL. To sum up, AtRRP6L1 may function in maintenance of the levels of H3K27me3 at the *FLC* locus by regulating ASL [92]. This kind of lncRNA-mediated chromatin modification also occurs in animals. In addition, identification of the lncRNAs involved in the regulation of flowering time in plants also revealed the complex regulatory mechanisms of lncRNAs.

### 4.5. LncRNAs in RdDM

RdDM is a plant-specific pathway involving *de novo* DNA methylation, as shown by many examples in model plants, such as maize and *Arabidopsis* [104]. RdDM requires the plant-specific RNA polymerases IV (RNAP IV) and V (RNAP V) to produce the required 24-nt siRNAs and lncRNAs [105,106]. The RdDM pathway regulates biological processes such as allelic crosstalk and antiviral defense by maintaining the silencing of transposons and repetitive elements [104]. Single-stranded siRNA precursors produced by RNAP IV are processed into 24-nt siRNAs by RDR2 (RNA-dependent RNA polymerase 2) and DCL3 (Dicer-Like 3). RNAP V produces the lncRNAs that act as scaffolds to guide the AGO4-siRNA complexes to the target loci, thus directing the *de novo* cytosine methylation in the complementary sequences [104].

In *Arabidopsis*, the modulation of methylation by the RdDM pathway regulates gene expression under stress conditions. *NRPD1* and *NRPE1* encode the largest subunits of RNAP IV and RNAP V, respectively. Heat stress induces the expression of *NRPD1* and *NRPE1*, and causes increased methylation of the SINE-like retrotransposon AtSN1, indicating that the RdDM pathway mobilizes under heat stress to strengthen DNA methylation [107]. In rice, psi-LDMAR siRNA processed from the LDMAR region associates with the DNA methylation level of the *LDMAR* promoter, thus the RdDM pathway participates in regulating photoperiod-sensitive male sterility by affecting the expression of the lncRNA LDMAR [79,80]. In maize, research on the *mop1* (mediator of paramutation 1) mutant, which affects a gene orthologous to *Arabidopsis* RNA-dependent RNA polymerase, indicated that mop1 is essential for establishment and maintenance of silencing of several genes [108,109,110]. These findings show that the RdDM pathway also has significant roles in epigenetic regulation, particularly in plants. All the mechanisms above increase our understanding of the roles of lncRNAs in plants and offer new ideas for the exploration of novel pathways related to lncRNAs in plants.

### 4.6. Genome-Wide Identification of Plant LncRNAs

Whole-genome tiling-array and RNA-seq analyses have revealed the complex transcriptional scenery in eukaryotes [111]. For example, in plants, as in animals, emerging research has used bioinformatics, next-generation sequencing, and other methods to identify lncRNAs. With the rapid development of genomic sequencing techniques, genome-wide identification of lncRNAs has been conducted by cDNA/EST *in silico* mining [112,113], epigenetic signature-based approaches [114,115], whole genome tiling-arrays, and other bioinformatics methods [116,117]. Typical procedures for computational identification of lncRNAs involve choosing transcripts without complete ORFs or with short ORFs [118,119,120,121,122]. However, such methods have some disadvantages. For example, many bioinformatics pipelines, such as tiling arrays, require existing genome sequence, hampering analysis of some species that lack annotated genome sequence. Also, low sensitivity may prevent the detection of rare transcripts. The drawbacks of tiling arrays can be overcome by RNA-seq, and new techniques are emerging to identify the complexity of the transcriptome with high precision [117]. For example, implementation of SVM (support vector machines) approaches allows researchers to distinguish whether transcripts encode proteins, overcoming a deficiency of typical methods [123,124] by using several criteria to assess the completeness, quality, and homology of ORFs, and thus improve the determination of the likelihood that a given transcript encodes a protein [125].

Emerging techniques and information from animal systems will enable the identification of more lncRNAs in plants. Determining the functions of lncRNAs in plants will provide breakthroughs in our understanding of many outstanding issues, both in plants and in animals. Genome-wide identification of lncRNAs has been conducted in *O. sativa* [116], *A. thaliana* [118,119,126], *M. truncatula* [122], *Z. mays* [125], and others. As mentioned above, in maize, a bioinformatics pipeline using Python and SVM tools identified 1802 lncRNAs based on full-length cDNA sequences. About 60% of the lncRNA sequences were annotated as precursors of small RNAs [125]. To acquire a more comprehensive understanding of lncRNAs in maize, Li *et al.* [127] combined public ESTs with RNA-seq datasets and whole-genome sequence annotation to identify 20,163 putative lncRNAs from 30 different experiments. Of these, they considered 1704 as high-confidence lncRNAs and over 90% as potential precursors of small RNAs [127]. LncRNAs identified in maize revealed that majority of the lncRNAs are precursors of small RNAs, an observation that provides insight on the relationship between small RNAs and lncRNAs. The diverse functions of the discovered lncRNAs indicate that they have many potential roles in every aspect of plant life. Also, many lncRNAs may show tissue-specific expression, which shows that lncRNAs influence diverse plant developmental processes. Compared to other model plants, like *A. thaliana* and *Z. mays*, the roles of lncRNAs in forest trees remain largely unknown. In *Populus tomentosa*, we identified novel lncRNAs differentially expressed in the xylem of different types of wood produced under mechanical stress [128]. Overall, we identified 1377 putative lncRNAs by computational analysis from RNA-seq data, of which 776 putative lncRNAs showed differential expression. Moreover, we found lncRNAs target sixteen genes involved in wood formation, indicating the potential function of lncRNAs in wood formation. Thus, exploration of non-model species can provide additional insights on the functions of lncRNAs.

Research on plants has revealed the complex relationship of lncRNAs and miRNAs. On the one hand, lncRNAs, such as LDMAR, can act as the precursors of small RNAs. On the other hand, some plant lncRNAs can also function as the target mimics. Our study of poplar also detected multiple mechanisms of interaction of lncRNAs and miRNAs. We identified three lncRNAs as the precursors of four miRNAs, and 25 lncRNAs as the targets of 44 miRNAs. The network of lncRNAs, mRNAs, and miRNAs illustrates the complex potential regulatory roles of lncRNAs in forest trees.

Genome-wide identification of lncRNAs in animals and plants provides a new insight to explore the broadly diverse mechanisms of lncRNAs. Emerging techniques that identify large amounts of lncRNAs with different characteristics have produced substantial advances in our understanding. In plants, we can identify lncRNAs at the genome-wide level, test their different expression patterns, analyze their distribution and expression levels in different developmental stages and tissues, investigate the action of lncRNAs on their targets, and so on. Thus we can improve our understanding of the genetic mechanism of lncRNAs in plants and extend our knowledge of the roles of lncRNAs in all species.

### 4.7. LncRNAs in Responses to Biotic and Abiotic Stress

Plants encounter various stresses from the environment and pathogens and have evolved sophisticated mechanisms to survive these biotic and abiotic stresses. LncRNAs participate in the response to stress and genome-wide identification and transcriptome analyses have found lncRNAs that function in different stresses.

Long noncoding natural antisense transcripts (lncNATs), and antisense lncRNAs transcribe from the antisense strand of protein-coding genes. The sense-antisense transcript pairs composed of lncNATs and their sense transcripts regulate gene expression in response to abiotic and biotic stresses [129,130]. Genome-wide analysis of NATs in *Arabidopsis* identified 37,238 sense-antisense pairs [131] that showed either concordant regulation, in which both members were regulated in the same direction, or discordant regulation, in which the members were regulated in opposite directions. Also, light regulated 626 concordant and 766 discordant lncNAT pairs in a developmental and spatial specific manner, indicating that these pairs participate in light responses. As expected, some of the NAT pairs identified in previous studies were not detected, which supported the point that lncRNAs are expressed in response to specific stresses or in different developmental stages. In addition, levels of these light-responsive NAT pairs were strongly correlated with the histone acetylation of the genes, suggesting a potential role of lncNATs in mediating histone modification for regulation of their sense transcripts in the light response [131].

In foxtail millet, genome-wide identification of ncRNAs was carried out by deep sequencing under drought stress [132] and found 584 lncRNAs that responded to simulated drought stress. Compared to the protein-coding genes, the shorter length and low expression of the lncRNAs agreed with results from previous work. PEG-induced water-deficit stress induced 17 lincRNAs (long intergenic ncRNAs) and 2 lncNATs at different expression levels. The weak correlation of levels of lincRNAs and their neighboring coding genes demonstrated that physical proximity may not be the only mechanism of lincRNA regulation [132].

Recent work also conducted genome-wide identification of drought-responsive lincRNAs in *P. trichocarpa* [133]. High-throughput RNA-seq was used to detect lincRNAs, including novel lincRNAs and those with low expression. In total, 2542 lincRNA candidates were identified from the *Populus* RNA-seq data, including 504 drought-responsive lincRNAs. RT-PCR showed that 8 of the lincRNAs are closely related to the drought response, 20 lincRNAs may function as target mimics, and 51 linRNAs may be targets of known *Populus* miRNAs [133].

In wheat, four lncRNAs, TalncRNA18, TalncRNA73, TalncRNA106, and TalncRNA108, isolated from ESTs correlated to stripe rust pathogen, participate in defense against stripe rust pathogen [134]. The expression of TalncRNA18, TalncRNA73, and TalncRNA106 increased during the early stages after inoculation with stripe rust pathogen in resistant genotypes. Even though the expression of TalncRNA108 decreased at early stages, its expression increased afterwards in resistant genotypes [134]. The up- and down-regulation of these lncRNAs in resistant genotypes after inoculation with stripe rust pathogen demonstrated that they may control the expression of genes related to defense against this pathogen.

In addition, Zhu *et al.* [135] identified a number of lncRNAs related to biotic stress using strand-specific RNA sequencing. In this study, they investigated the change of NATs and TARs (transcriptionally active regions) after infection of *A. thaliana* with *Fusarium oxysporum* and found 20 novel lincRNAs, 159 novel, intergenic TARs, 10 induced lncNATs, and 5 repressed lncNATs. The potential role of 10 lincRNAs in antifungal immunity was confirmed by examination of T-DNA insertion and RNAi lines. Five lincRNAs showed more rapid or more severe disease symptoms in T-DNA insertion and RNAi lines. In addition, promoter analysis of lincRNA in TARs with their adjacent genes indicates that some of the lincRNAs induced by *F. oxysporum* were the targets of transcription factors related to pathogen responses. This differs from the results observed in foxtail millet [132], which suggested that lincRNAs might affect their adjacent genes by physical proximity. LincRNAs may regulate gene expression through various pathways. Some lncNATs and their corresponding genes showed co-regulation, which demonstrated that these lncNATs likely affect the expression of proteins associated with disease resistance. The expression pattern and characteristics of these lncRNAs responding to *F. oxysporum* reveal their crucial role in pathogen immunity [135].

These findings in plants extend our understanding of the varied roles of lncRNAs. Finding new mechanisms of lncRNA function increases our awareness of lncRNAs, and also enlarges the network of known types of RNA. For example, the discovery of the miRNA regulatory mechanism in plants, in which lncRNAs act as target mimics of miRNAs, lays a foundation for practical applications of lncRNAs and improves our understanding of the network of lncRNAs and miRNAs. It is notable that the structure of lncRNAs may yield new insights into the function of lncRNAs. In *A. thaliana*, the HID1 lncRNA acts as a positive regulator of photomorphogenesis and HID1 under continuous red requires two of the four predicted stem-loops. No small RNAs derived from the stem-loops of HID1, indicating that the complex structure of HID1 is vital for its biological function [93]. The investigation of the structure of lncRNAs can be considered as a key part of the elaboration of lncRNA function.

## 5. Perspectives

Myriad lncRNAs have been identified in animals and plants with the development of high-throughput transcriptome sequencing. Computational methods have enabled researchers to predict the function, location, and classification of lncRNAs, and have produced remarkable results and revealed potential practical applications. The expression profiles of lncRNAs can be treated as biomarkers for some diseases, like *BACE1-AS* in Alzheimer’s disease. Some of the molecular functions of lncRNAs have been identified in plants. For example, plant lncRNAs can serve as molecular cargo in re-localization of key proteins. Also, lncRNAs can serve as molecular scaffolds to cause chromatin modifications and recruit protein complexes [136]. In plants, some of the epigenetic mechanisms of lncRNAs have also become clear [137] and the complex network of lncRNAs and miRNAs shows the potential regulatory roles of lncRNAs in plants. Thus, lncRNAs in plants can be considered as essential elements of gene regulation. The future study of lncRNAs will reveal a more complete picture of their functions, and the detailed information on this genomic “dark matter” will enable researchers to use lncRNAs in treating disease, improving agricultural production, and solving many outstanding mysteries in plants and animals.
